# The appearance and increase in the quantity and proportion of the clinical research coordinator’s service fee in drug clinical trial research fund and its impact on trial quality

**DOI:** 10.1186/s12962-021-00297-1

**Published:** 2021-07-12

**Authors:** Liran Chen, Zhimin Chen, Huafang Chen

**Affiliations:** 1 The University of Queensland, Brisbane, Australia; 2 Ningbo Hospital of Traditional Chinese Medicine, Zhejiang, China; 3 Office of Drug Clinical Trial Institution, The First Affiliated Hospital of Wenzhou Medical University, Nanbaixiang Street, Ouhai District, Wenzhou, 325000 Zhejiang China

**Keywords:** Clinical research coordinator, Site management organization, Clinical trial, Fund, Inspection, Quality

## Abstract

**Objective:**

The changes of absolute value and relative value of clinical research coordinator service fee and its influence on the quality of drug clinical trial were analyzed.

**Methods:**

This study compared the amount and structural changes of drug clinical trial costs in before 3 years and after 3 years of self-examination and inspection initiated by the China Food and Drug Administration, identified the increase number and composition of each individual cost of a clinical trial research funds which including clinical research coordinator service fee, investigator labor fee, subjects examination fee, subjects traffic subsidy, documents management fee, drug management fee, etc.

**Result:**

The most significant appearance of increase in volume and proportion was the clinical research coordinator service fee. From the initial few to the global multicenter tumor drug clinical trials RMB31,624 or 34.92% of the proportion and domestic multicenter tumor drug clinical trials RMB16,500, accounted for 33.74%.

**Discussion:**

It has become common for more money to be spent on clinical trials to be accompanied by improved quality, but the occurrence and continuous increase of clinical research coordinator service fee were divided into two aspects, On the one hand, the quality of clinical trials was promoted by the large amount of low-skill trivial work undertaken by clinical research coordinator; on the other hand, the quality of clinical trials was undermined by the fact that clinical research coordinator did too much treatment evaluation work that should have been done by the investigator.

**Conclusion:**

The clinical research coordinators’ access standards, pre-employment training and examination, job and performance evaluation, in addition to the SMO specification management and avoiding malicious competition between the industry, are important factors in the quality assurance of drug clinical trials.

## Background

On July 22, 2015, The China Food and Drug Administration issued The Announcement of Self-examination and Inspection of Drug Clinical Trial Data [1] and annotated 1622 drug registration applications list. As a thunder, it opened what the industry called the 7.22 storm. With “the most rigorous standards, the most strict supervision, the most severe punishment, the most serious accountability”, the most stringent drug registration self-examination and inspection in history was launched.

In the bulletin of 7.22, CFDA requires all pharmaceutical companies who have submitted an applicant of drug registration and are waiting to be approved must carry out self-examination on the clinical trials in accordance with the 《Good Clinical Practice of Pharmaceutical Products》 [2] and the clinical trial protocol, so as to ensure the authenticity and reliability of clinical trial data and the integrity of relevant evidence. The application can be withdrawn if any problem was found in pharmaceutical companies’ self-examination. If the pharmaceutical company continues to apply after the self-examination and the authenticity problem is found in the CFDA inspections, all application of this pharmaceutical company will not be accepted within 3 years. If a drug clinical trial medical institution practices fraud, its qualification shall be revoked.

In about four months from late July to early November 2015, as the sponsors of clinical trials and registration applications, pharmaceutical company began to implement self-examination. Before that, CFDA did not carry out extensive clinical trial inspection, and did not issue detailed inspection standards for reference. After some struggles, 1622 pharmaceutical companies may lack confidence in the quality of their clinical trials, or may be in order to avoid greater risks, exceeding 80% sponsor chose to withdraw. On November 10, 2015, CFDA issued The Announcement of key points for On-site Inspection of Clinical Trial Data [3]. Since then the on-site inspection of clinical trials officially started in China on a large scale. Every drug registration application must go through on-site inspection of clinical trials to prove that the trial data are true and reliable before it can be approved.

In this storm, some pharmaceutical companies were disheartened after withdrawing, and some pharmaceutical companies were embarrassed after CFDA announced the verification results and suspected of fraud. Before July 22 2015, the investment in drug clinical trial was less, the quality level was uneven, the price war was disordered, and “Many, small, scattered and disordered” were common in China’s pharmaceutical industry. Many enterprises do not fulfill their responsibilities for drug clinical trials, and they do not control the drug clinical trial process and quality enough, so they submit the defective trial dates to CFDA. Through self-examination and inspection, CFDA forced pharmaceutical enterprises to recognize their responsibilities in clinical trials, as a sponsor of clinical trial, if they do not improve the quality of clinical trials, they will be trapped in the old thinking and eliminated.

Clinical trials are the highest investment in all aspects of new drug research. Clinical trials need to collect data on the efficacy and safety of a large number of subjects after using drugs, it is necessary to demonstrate that the test drug is at least as effective as a placebo or equivalent to a marketed control drug. When the drug clinical trials are conducted in the medical institutions, the sponsor will need to pay the investigator, as a doctor, who diagnosed and treated the subjects and collected data according to the trial protocol, and need to pay labor costs to clinical research coordinator (CRC). CRCs have become increasingly common among clinical trial teams in recent years, whose job is to assist investigator with simple and tedious work, but not medical diagnosis and treatment. The presence of a CRC has become needful in order to conduct quality academic research [4]. CRC is the person with whom subjects interact the most [5]. A key role in the infrastructures is played by the CRC, a key figure able to manage the workflow required, placing himself as a reference for the coordination of the various activities and professional figures involved [6]. Except for the investigators and the CRCs, the sponsor is required to pay all the trial related expenses of the subjects accordance with the ethical requirements [7], including cost of all kinds of examine, traffic subsidy, buying control drugs, and so on. If the sponsor wants to implement high-quality and standard clinical trials, it must invest a lot of manpower and spend a lot of money. After 7.22, great changes have taken place in the field of drug clinical trials in China, among which the biggest change is the improvement of quality, and the increase in cost of clinical trials is an important change, because improvement of quality requires significant investment of human and financial resources, along with the quality improvement, the change of cost is inevitable.

### Objective

The purpose of this study is to analyze the changes in quantity and structure of clinical trials costs before and after 7.22 storm and explore the corresponding relation between cost change and quality improvement.

## Methods

There are different visit procedures and difficulty levels in different drug clinical trial protocols. In recent years, cancer drugs have been frequently involved in clinical trials, because the input costs of similar drugs are comparable [8]. For example, we can analyze the change in the amount and composition of the clinical trials cost. In this study, the data come from the First Affiliated Hospital of Wenzhou Medical University, Tianjin Medical University General Hospital and Xiehe Hospital Tongji Medical College Huazhong University of Science and Technology. Since multi-center drug clinical trials are generally signed contracts at the same price at every site, the three hospitals can represent the average cost level over the same period in China.

The number and structural changes of drug clinical trial costs in before 3 years and after 3 years of self-examination and inspection organized to deploy by the China Food and Drug Administration were analyzed, and determined the increase number and composition of each individual cost of a clinical trial research funds. The costs of drug clinical trial include investigator labor fee, subjects examination fee, subjects transportation subsidy, documents management fee, drug management fee, CRC service fee, etc. (Table 1). The unit of measurement is RMB (RMB yuan). Data analysis was performed using Statistical Package for the Social Sciences (SPSS) software version 18 (SPSS Inc., Chicago, IL, USA). Descriptive information was presented as mean and percentage.

**Table 1:** Costs structural of drug clinical trial involved in the agreements signed by the hospital before and after 7.22 in China

Cost structure at different times		Purpose of cost
Before 7.22	After 7.22	
Investigator fee	Investigator fee	The service cost of the Investigator’s diagnosis, treatment and evaluation
Examination fee	Examination fee	Be charged for subjects’ imaging, laboratory tests of biological samples etc
Transportation subsidy	Transportation subsidy	The cost of transportation for participants in the clinical trial
Documents management	Documents management	The cost of save trial files for a long time as required by the regulations
	Drugs management	The service costs for the administration of trial drugs
	CRC fee	The cost of service for personnel assisting the investigator

This study focuses on the costs involved in the agreements signed by the hospital during innovative drug phase II, III and generic drugs random controlled clinical trials, and make little analysis on the monitoring, audit and tax input of the sponsor. It does not involve the costs of phase I clinical trials, clinical trials data management statistics, cold chain logistics or researcher meetings.

Cancer drugs are common varieties of drug clinical trials in recent years, so the cost of cancer drugs clinical trials is representative. We take the cost of lung cancer drugs which includes 18–20 visits excluding the lifetime visits and unscheduled visits, for example, compare the cost of clinical trials before and after July 22, 2015.

## Result

**Fig. 1:**
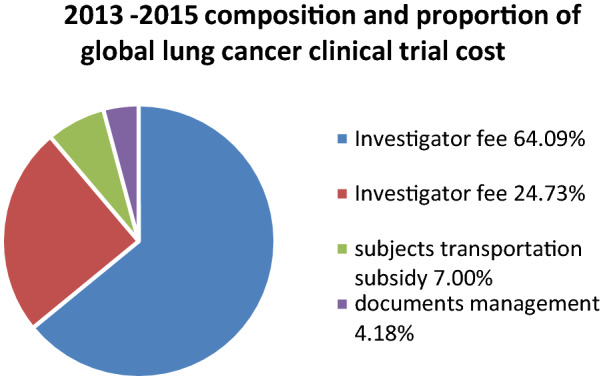
2013–2015 composition and proportion of global lung cancer clinical trial cost

The composition and proportion costs of clinical trial with international sponsors within China 3 years before July 2015 within China (taking lung cancer drugs as an example) as follow, the average costs from 2013 to2015 were showed as Fig. 1 as follows: the investigator fee was 30697or 64.09% of the proportion, the subjects examination fee was 11,847 or 24.73% of the proportion, the subjects transportation subsidy was 3355, accounting for 7.00% and the documents management fee was 2000, accounted for 4.18%.

**Fig. 2:**
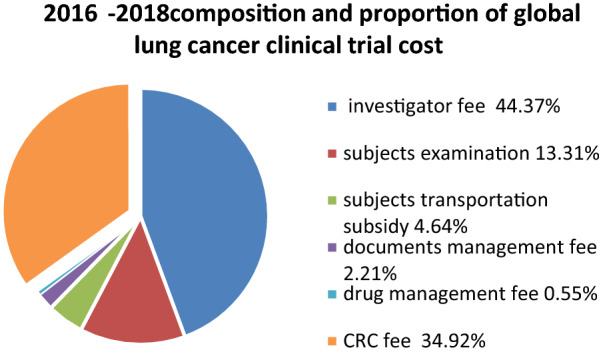
2016–2018composition and proportion of global lung cancer clinical trial cost

The Composition and proportion costs of clinical trial with international sponsors within China 3 years after July 2015 within China as follow, the average costs from 2016 to 2018 were listed in Fig. 2: investigator fee was 40,187 or 44.37% of the proportion, the subjects examination fee was 12,053 or 13.31% of the proportion. The subjects transportation subsidy was 4200 or 4.64% of the proportion, documents management fee was 2000 or 2.21% of the proportion, drug management fee was 500 or 0.55% of the proportion. It is worth asking that the CRC fee was 31,624 or 34.92% of the proportion.

**Fig. 3:**
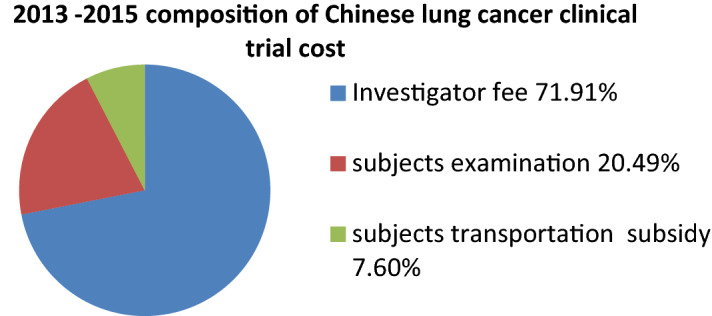
2013–2015 composition of Chinese lung cancer clinical trial cost

The **c**omposition and proportion of the costs of Chinese clinical trial within China 3 years before July 2015 (taking lung cancer drugs as an example) were showed Fig. 3. The average costs from 2013 to 2015 as follows: the investigator fee was 18,930 or 71.91% of the proportion, the subjects examination fee was 5395 or 20.49% of the proportion, the subjects transportation subsidy 2000, accounting for 7.60% and no documents management fee was charged.

**Fig. 4:**
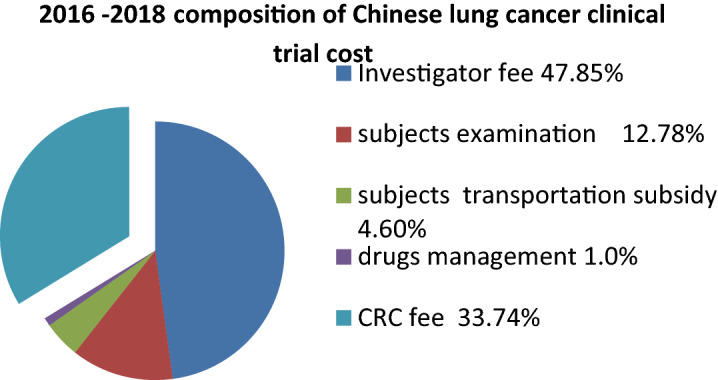
2016–2018 composition of Chinese lung cancer clinical trial cost

The **c**omposition and proportion of Chinese clinical trial cost 3 years after July 2015 within China as follows, the average costs from 2016 to 2018 are shown in Fig. 4: the investigator fee was 23,400 or 47.85% of the proportion; the subjects examination fee was 6252 or 12.78% of the proportion; the subjects transportation subsidy 2250, accounting for 4.60%; the drugs management fee was 500, accounted for 1.00%; the CRC fee up to 16,500, accounted for 33.74%, and no documents management fee was charged.

Comparing Figs. 1, 2, 3 and 4, the investigator labor fee and CRC service fee in global multiple-center clinical trial is significantly higher than that fees of China. Besides the gap of sponsor economic power, the global multiple center clinical trials focus on innovative drug while the majority of China clinical trials are generic drugs clinical trials.

The changes presented are as follows when above each individual cost of a clinical trial research funds were analyzed.

Investigator fee: the increase in the investigator fee of global clinical trial cost is not obvious, and eliminating inflation the amount shows little change in Fig. 1. Figure 2 shows that the cost of the Chinese multi-center site grew notably by 30–40% after 7.22.

CRC fee: the CRC fee shows the most significant change. There were few CRC fee expenditures before July 22 2015, but fees increased starting year by year from 2016 to 2018. Figure 2 shows that the mean of CRC fee for multi-center clinical trial for cancer drugs internationally from 2016 to 2018 was 31,624, and proportion was 34.92%. Figure 4 shows that the mean CRC fee associated with the Chinese multi-center clinical trial for cancer drugs from 2016 to 2018 was 16,500, and proportion was 33.74%.

Drugs management fee: The 7.22 promoted the standardized management of drugs in GCP central pharmacy and the process of drug clinical trials, due to the low amount of its fee, it represented only approximately 1% of the total investment in drug clinical trials.

Documents management fee: there are some differences between hospitals in terms of Chinese and global clinical trials. According to the Good Clinical Practice of Pharmaceutical Products [SFDA order No. 3, Effective on September 1, 2003] Article 52: “The investigator shall keep the documents of the clinical trial for five years after the completion of the trial.” [2] However, after the completion of drugs clinical trial with international sponsors, the documents generally must be kept for 15 years. The documents global trial was preserved freely with reference to China’s domestic the first 5 years when it was completed, the charge standard of 6–15 years, on the basis of the cases amount and number of documents, hospitals charge 1500–5000 every year. After 5 years, the documents from completed clinical trial in China can also be handed over to a qualified third party specified by the sponsor and paid to the third party.

Other related changes of costs were not covered by the clinical trial agreement before and after the 7.22, such as the cost for monitor, audit.taxes. After 7.22, along with CFDI inspect strongly in concomitant drugs, adverse events (AE) and serious adverse events (SAE) of subjects, monitor need to check the Hospital Information System (HIS), Laboratory Information System (LIS), picture archiving and communication systems (PACS) of hospital in the process of monitoring, to ensure that a combination drug, especially a combination drug prohibited by the protocol, AE and SAE are recorded Completely without omission. In recent years, the risk-based monitoring advocated by the sponsor has improved the monitoring efficiency certain to some degree, but in general, after 7.22, monitoring will spend 15–20% more time in the hospital, and the corresponding labor cost will increase 15–20%. In addition, the State Administration of Taxation changed the Value Added Tax (VAT) rules at 2017 in China, and the clinical trial cost in invoice billing system was within the scope of tax, when sponsors remit money to hospitals, the added 6% tax must be paid Thus, there was a 6 percent increase in the total amount of money the sponsor had to pay. Of course, the tax cost has nothing to do with the 7.22 storm.

## Discussion

Following the 7.22 storm, along with CFDA’s stringent requirements for clinical trial quality, the input of human and financial resources increased. There have been some noticeable changes, such as the emergence of Site Management Organization (SMO), the increase in clinical trial costs, the increase in audit companies and the centralization of drug management in research site.

Both the CRCs and audits, sponsors are required to pay for their labor. The increase in CRC fee was the most rapid and significant change associated with the cost of clinical trials, which is clearly shown in Figs. 1, 2, 3 and 4. Improving the quality of clinical trials requires a great deal of fine work, and it takes a lot of human resources to accomplish these works. An SMO provides clinical study coordinator services, and sends CRC to the hospital to assist in the implementation of the clinical trial and performs the nonmedical judgment duties authorized by the Principal investigator (PI). SMOs began to develop in 2008 in China and remained small scale until July 22 2015. Then, the number of SMO expanded rapidly, and the staff of SMOs increased, with one SMO growing to include nearly 600 CRCs within one year of its establishment. After 7.22, third-party audit institutions sprung up successively which provide clinical trial training, consulting services, and audit comprehensively on-site of clinical trial data and records. Their independent examine mode makes the division of labor refined and specialized. The audit company usually sends 5 persons to a site for 2–3 days on-site audit, and the sponsor needs to pay the audit company a fee of 80–100 thousand yuan. This part of the cost was not listed in the cost list in the result part because it was paid to the audits by the sponsors without going through the hospital.

The increase in the investigator labor fee is divided into explicit growth and implicit growth. Explicit growth can be clearly shown in Figs. 1, 2, 3 and 4. We can see that the sponsor paid more money for investigators’ works in order to improve the quality after 7.22, especially in multi-center clinical trial of China. Remove explicit growth, implicit growth refers to the addition of the CRCs, in that part of the nonmedical judgment work that done by CRC. The Investigators' labor fees have increased in disguised form because of the decrease in their workload.

Drug management fee and quality control fee also appeared. The drug management fee is small, and each subject is generally will charge a fee of 200–500 yuan according to the complexity level of the clinical trial, this method has played a positive role in regulating the management of drugs in the implementation of drug clinical trials. Before 7.22, the drug clinical trial research institutions charged lower quality control fees, and some institution began charging quality control fees after 7.22. The fees charged by agencies vary, and the overall amount is small. The fees are earmark for this specified purpose and therefore play a relatively important role in quality control [8].

Cost-effectiveness analysis is an essential and commonly used approach for such priority setting in healthcare [9]. However, high investment and high cost do not necessarily guarantee high quality. The price of clinical trials showed a sharp increase in nearly years. The quality of clinical trials also undergoes obvious change, but there is no direct correspondence between them [10]. There is a saying in the industry about the cost and quality of clinical trials: high costs do not necessarily mean high quality, but low costs certainly cannot yield high quality. The consensus in the industry is that low cost cannot guarantee the quality of clinical trials, and any step is requires a budget for labor and material resources. Most of the cost increases in clinical trials are positively correlated with quality improvements [11], but there are many factors influencing quality, so it is not simply a matter of increasing investment that can improve quality, for example, investment in CRC.

The increase in costs caused by the booming development of SMO and the improvement in the quality of clinical trials should be viewed in two aspects. The CRC service fee paid by the sponsor to the SMO accounts for approximately 30% of the total investment in clinical trials, which cannot be underestimated. The intervention of the CRC has undeniably provided convenience and some assistance for investigators to improve the quality of clinical trials. In contrast, trials initiated by the sponsor required 65% of the services during project set-up and conduct phase, which comprises support by study nurses or CRCs in the on-site management [12]. When resources permit, it is also helpful to hire a manager or coordinator who supervises the program by overseeing quality assurance, staffing, budgeting, and site audits. CRCs will work closely with the primary investigator to ensure that all investigator responsibilities are being met [13]. However, there are also disadvantages associated with the deficient abilities of the CRC, the investigator absence of the management of clinical trials and the excessive dependence of the investigator on the CRC.

After 7.22, the research institutions that undertake clinical drug trials asked CRCs to facilitate trials at the time of signing the agreement with the sponsor. The CRC agreement is a separate triple agreement is generally independent from the main clinical trial agreement and is signed by the sponsor, SMO and the hospital (research institution). Since the financial management of Chinese public hospitals does not allow payment to be easily sent to businesses such as SMOs, it is stipulated in the triple agreement that the sponsor shall remit money to the SMO, and the SMO shall send a CRC with a relevant professional background and industry training to the hospital to assist with the investigator’s work. The principal investigator authorizes and assigns the CRC tasks of nonmedical judgment, and the CRC is authorized to do ancillary work, the responsibility for clinical trials remains with the principal investigators and subinvestigators, when the CRC is incompetent, hospitals have the right to require the SMOs to replace him or her with a competent CRC.

Newly registered SMOs have sprung up in recent years, and the shortage of CRC talent has led to a war between SMO companies. CRCs frequently job hop, and an impetuous atmosphere is spreading within the industry, which results in a profit-oriented short-term development model among SMOs. The training process before a new CRC takes up the post can be shortened or even omitted. Another problem is that the management of CRCs by SMOs is regulated via long-distance supervision. The CRCs work in various hospitals in different districts, and the SMO functions in the registration district, which also makes supervision difficult. The lack of industry training [14] and supervision are obvious shortfalls in the SMO industry which show a rapid growth in quantity, not quality. The majority of CRC practitioners are mostly undergraduate or even community college graduates in pharmacy or nursing, and their professional skills are poor. In addition, most of them enter the CRC industry immediately after graduation, with no working experience and insufficient professional knowledge accumulation [15]. In addition to the inadequate training of SMO companies, many incompetent CRCs are sent to hospitals to perform CRC functions. Investigators and sponsors (upervising and urging the SMO) should take responsibility to ensure that CRCs have adequate training in areas that we have highlighted [16].

From the perspective of the hospital, the investigators consider themselves very lucky if an excellent CRC is sent to their site; a CRC of average quality is normal; and even a incompetent CRC is better than none. In other words, the investigators dare not expect a good CRC, considering that any CRC is better than none. While the CRCs have low-quality skills, they have plenty of time to assist the investigators; on the other hand, the investigators are too busy with clinical work and his patients to focus on the clinical trials [17]. In such a state, CRCs perform the duties of many of the investigators and even the principal investigators. The principal investigators’ work is to sign for authorizations, then engage in clinical work, hand over the clinical trial work to CRC, When the clinical trial forms is need to be signed during the subsequent trial, these forms are all signed by the primary investigator and the investigator after the CRC has filled in and prepared. Most of the time, the contents of the signed forms are not asked before the primary investigator and the investigator hurriedly sign them. Another common phenomenon concerns the CRCs’ use of the investigators’ account and password authorized by the sponsor for logging into the EDC (electronic data capture system). when CRC used the investigators’ account and passwords to fill out numerous forms, the investigators ignored the review before the investigator and primary investigator signed it, so the process and date completed by the CRCs have not been verified by investigators blind signatures, error-ridden process and content and the investigators’ leaking of EDC account passwords to CRCs mean that the monitoring of the clinical trials may not cover the whole process and entail hidden dangers in clinical drug trials [18].

The long-distance supervision of the CRC by the SMO underachieved in their job, and there is a lag in the evaluation of CRC work quality. In that situation, timely and effective feedback is lacking. Once a CRC’s incompetence is discovered, the effects on the quality of the clinical trial and the trial schedule are irreversible. The long-distance supervision by the sponsor and the SMO cannot effectively control the quality of CRCs. If the hospital office managers and investigators of clinical trials find that a CRC does not meet the quality requirements and a replacement of CRC is required, the hospital staff must consider the time and opportunity costs of CRC turnover. Hospitals are always at risk, for example, the replacement CRC may also be incompetent; it takes time for the new CRC become familiar with the work; interactions with different CRCs may also lead to mutual buck-passing due to the work handover when problems arise.

Given the unsatisfactory state of the CRC industry, there have been other attempts, but they have not worked very well. Apart from the SMOs providing CRCs, individual hospitals, such as Zhongshan Hospital Affiliated of Fudan University, Beijing 301 Hospital, Hunan Cancer Hospital and Cancer Hospital Affiliated of Sun Yat-sen University, established their own CRC teams to assist with clinical trials. Their purpose is to create a high-level and stable CRC team that can assist investigators in accomplishing high-quality clinical drug trials, turning from the model of outsourcing CRC services from SMOs to the model of training, employing and supervising CRCs within the hospital itself. Due to the limitation of traditional staffing model in Chinese public hospitals, the hospitals’ own CRCs account for a small proportion of the total number of CRCs currently employed by SMOs. Each model for supplying CRCs, either outsourcing from SMOs or providing CRCs within hospital, has advantages and disadvantages. CRCs sent by SMOs can cover clinical trial sites in cities across the country, but the personnel sent by SMOs to hospitals need to undergo a process of familiarization and be accepted by hospitals. A CRC team within a hospital has many advantages in undertaking local work, but it cannot support the clinical trial needs of other hospitals.

The lack of universally recognized industry regulations on CRCs is the key in current China. Similar patterns have emerged in other countries, Cagnazzo’s paper [6] pointed out that there is no yet an institutional recognition of the professional figure, neither a specific economical agreement within the National Health System. Some professional associations are actively formulating industry standards and qualification certifications for CRCs. For example, the Professional Committee of Pharmaceutical Clinical Trials of Guangdong put forward the Guangdong consensus on CRC management, and the Alliance of Pharmaceutical Clinical Trial Institutions issued CRC industry guidelines. Despite the efforts of the sponsors, hospitals and even SMOs, there are no clear and commonly accepted operational standards.

From a positive perspective, 7.22 has opened up a new situation of quality requirements for clinical trials, but much remains to be accomplished in the gradual and ongoing process of improving clinical drug trials. Only cooperation and continuous collaborative efforts can lead to progress.

## Conclusion

A cost increase of drug clinical trial is necessary for quality improvement, but it is not a sufficient condition. Every relevant aspect should be well regulated and managed, which in turn is economically important particularly in developing countries with a shortage in financial resources [19]. If one aspect is neglected or incomplete, the cost increase is not positively correlated with quality improvement, and certain factors that raise prices can improve quality in some ways, while obstructing quality improvement in other ways.

The CRC currently has undertaken a large number of basic works and has become an indispensable role in clinical trials. The CRC access standards, Pre-employment training and examination, job and performance evaluation, in addition to the SMO specification management and avoiding malicious competition between the industry, are important factors in the quality assurance of drug clinical trials.

## Data Availability

Data and materials are available.
